# The Role of Vascular Endothelial Growth Factor in Metastatic Prostate Cancer to the Skeleton

**DOI:** 10.1155/2013/418340

**Published:** 2013-12-12

**Authors:** Emma Roberts, Davina A. F. Cossigny, Gerald M. Y. Quan

**Affiliations:** ^1^Spinal Biology Research Laboratory, University of Melbourne, Department of Surgery, Austin Health, P.O. Box 5555, Heidelberg, VIC 3084, Australia; ^2^Department of Spinal Surgery, Austin Health, P.O. Box 5555, Heidelberg, VIC 3084, Australia

## Abstract

Despite the clinical implication and high incidence of bone and spinal metastases, the molecular mechanisms behind prostate cancer metastasis to bone and spine are not well understood. In this review the molecular mechanisms that may contribute to the highly metastatic phenotype of prostate cancer are discussed. Proangiogenic factors such as vascular endothelial growth factor (VEGF) have been shown to not only aid in the metastatic capabilities of prostate cancer but also encourage the colonization and growth of prostate tumour cells in the skeleton. The importance of VEGF in the complex process of prostate cancer dissemination to the skeleton is discussed, including its role in the development of the bone premetastatic niche, metastatic tumour cell recognition of bone, and bone remodeling. The expression of VEGF has also been shown to be upregulated in prostate cancer and is associated with clinical stage, Gleason score, tumour stage, progression, metastasis, and survival. Due to the multifaceted effect VEGF has on tumour angiogenesis, tumour cell proliferation, and bone destruction, therapies targeting the VEGF pathways have shown promising clinical application and are being investigated in clinical trials.

## 1. Introduction

The five-year survival rate for prostate cancer is extremely high when confined to the prostate, but in the presence of metastatic disease it is reduced to 33% [1]. In Australia, prostate cancer contributes to almost 5% of all registered male deaths [2] of which more than 80% will have developed spinal metastases during the course of their disease [3–5]. Once cancer metastasizes to bone and the vertebral column, patients often experience intractable pain and neurological deficit due to pathological fractures, spinal instability, and metastatic epidural spinal cord compression. The neurological sequelae include sensory disturbance, motor weakness, paralysis, and incontinence, leading to decreased function, inability to ambulate and impaired quality of life [5]. Treatment options include radiotherapy, hormonal therapy, chemotherapeutic agents such as docetaxel, cabazitaxel, sipuleucel-T and abiraterone acetate, and decompression and stabilization surgery [6, 7]. These modalities may be able to extend survival rates but are all predominantly palliative, with median survival time limited from one to two years from the onset of metastases [5, 6]. Despite the clinical implication and high incidence of spinal metastasis, the molecular mechanisms behind prostate cancer metastasis to bone and the spine are not well understood. Vascular endothelial growth factor (VEGF) is well known to be potent stimulator of angiogenesis in both physiological and pathological conditions and is highly expressed in most solid tumours, including prostate cancer. This review discusses the role of VEGF in tumour angiogenesis and bone destruction in metastatic prostate cancer to the spine.

## 2. VEGF and Its Receptors

**Figure 1:**
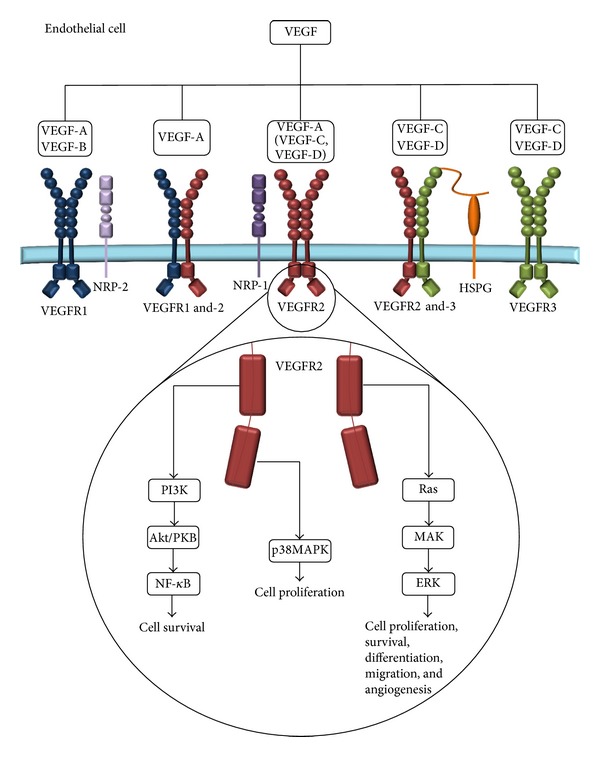
VEGF receptor binding. The five mammalian vascular endothelial growth factors (VEGF-A-D) bind to the receptor tyrosine kinases, VEGF receptor (VEGFR1-3 and co-receptors HSPG, NRP-1 and NRP-2). VEGFR-binding leads to the formation of homodimers and/or heterodimers. Proteolytic cleavage enables VEGF-C and -D to bind VEGFR-2 forming a homodimer. The binding and activation of VEGFR-2 lead to downstream signaling of the PI3 K, MAPK, and Ras pathways which promote cell survival, proliferation, differentiation, migration, and angiogenesis.

VEGF is a ligand of the VEGF tyrosine kinase receptor superfamily and includes VEGF-A, -B, -C and -D, with splice variants of VEGF-A resulting in several different isoforms [8, 9]. The VEGF family ligands bind to tyrosine kinase receptors VEGFR1, VEGFR2, and VEGFR3 (Figure 1), each receptor containing an extracellular domain of approximately 750 amino acid residues, arranged within seven immunoglobulin-like folds [10]. Additionally, heparin sulphate proteoglycans (HSPGs) as well as neuropilins (NRP-1 and NRP-2) can act as coreceptors for VEGF and promote VEGFR activation [11, 12]. Each VEGF family member binds with differential affinity for their receptors; for example, VEGFR2 is primarily activated by VEGF-A and VEGFR3 is only activated by VEGF-C and -D. Upon specific VEGF binding, the three VEGF receptors induce receptor dimerization and autophosphorylation leading to downstream signaling via a number of secondary messengers including several protein kinases and phosphatases that support a proangiogenic phenotype [10–12]. Important pathways include the phosphoinositide 3-kinase/Protein Kinase B/NF-*κ*B pathway that promotes cell survival, the mitogen-activated protein kinase (MAPK) pathway that promotes cell proliferation, and the Ras/extracellular signal-regulated kinase (ERK) pathway that promotes cell proliferation, survival, differentiation, migration, and angiogenesis. Through these signaling pathways each of the VEGF family provides different actions, with VEGF-A activation of VEGFR2 representing the major mediator of angiogenesis induction [13–15].

There are many factors that influence and regulate the VEGF/VEGFR pathway, including local environmental hypoxia and various hormones, growth factors, and cytokines. Hormones such as androgens upregulate stromal cell and malignant cell VEGF production and angiogenesis, enhancing prostate cancer growth [16–18]. As such, hormone withdrawal has been shown to inhibit VEGF expression as well as angiogenesis in prostate cancer patients while inducing apoptosis in these cells [19]. Growth factors such as PDGFs, TGF-*β*1, and IGFs also have a significant impact on the VEGF/VEGFR pathway by inducing the transcription and secretion of VEGF [18, 20, 21]. Cytokines such as TNF-*α*, IL-6, and IL-8 have also been shown to induce VEGF signalling to promote angiogenesis and tumourogenesis [22, 23]. IL-6 and IL-8 are also involved with the PI3 K/Akt/NF-*κ*B pathways as well as the MAPK pathway of VEGF signalling [24, 25].

## 3. VEGF and Angiogenesis

Angiogenesis is the growth and development of new blood vessels and is necessary to supply nutrients and maintain homeostasis in the tissues of the body [12]. Normal angiogenesis is tightly regulated by inducers and inhibitors of endothelial growth and is established from preexisting vessels, which develop ordered and predictable vasculature [26]. The actions of VEGF affect numerous cell types, thus enabling a multifaceted response. Initial activation of VEGF promotes the secretion of proteolytic enzymes to degrade the basement membrane and extracellular matrix whilst also aiding in the proliferation and migration of endothelial cells to form immature vasculature [13, 26]. VEGF also maintains newly formed vessels by inducing the expression of Bcl-2 and A1 anti-apoptotic proteins that promote cell survival, whilst activating colony formation by attracting mature subsets of granulocyte macrophage progenitor cells [27, 28]. VEGF-A exhibits vast up-regulation under hypoxic conditions whereby hypoxia-inducible factors (HIFs) stabilize and bind to specific promoter elements present in the promoter region of VEGF-A [11]. VEGFR1 and VEGFR2 are also directly regulated by HIFs [11].

In cancer, alterations in this balance of inducers and inhibitors in favour of angiogenesis can stimulate an “angiogenic switch”, via overexpresson of pro-angiogenic factors such as VEGF by tumour cells and tumour-associated stroma [26, 29]. Hypoxic conditions activate the uptake of VEGF and other growth factors and induce the growth of neovasculature, allowing the tumour cells to gain access to oxygen and nutrients [26, 29–31]. Indeed, the induction of angiogenesis has been shown to correlate with the invasive properties of tumours and is associated with poor prognosis [32]. Along with tumour vascularization, activation of genes governing the disruption of cell to cell adhesion and cell motility enables proliferation of primary tumour cells as well as allowing detached cells to disseminate throughout the circulatory system [28]. In benign prostate glands, VEGF expression is mainly confined to the basal cell layer and has weak levels of VEGF binding, while in prostate tumours VEGF is upregulated and found beyond this layer, including neoplastic secretory cells [33].

## 4. VEGF and Skeletal Metastasis

During the normal development of long bones and vertebrae or bone repair, growth and remodeling of bone formation occurs through osteogenesis [34]. A balanced state is created through the continuous and integrated processes of bone formation and deposition by osteoblasts and bone and mineralized matrix resorption by proteolytic enzymes and hydrochloric acid secreted by osteoclasts, derived from the haematopoietic stem cells of the bone marrow [35, 36]. It is a complex process dictated by growth factors and cytokines including fibroblast growth factors (FGFs), platelet-derived growth factor (PDGF), transforming growth factor-*β* (TGF-*β*), and bone morphogenetic proteins (BMPs) [34]. VEGF is expressed by osteoblasts and has autocrine and paracrine effects including chemotactic migration, proliferation, and differentiation of osteoblasts, as well as stimulating the formation, survival, and resorptive activity of osteoclasts. It is essential for normal angiogenesis and appropriate bone repair and mineralization in response to bone injury [37]. *In vivo, *absence of VEGF leads to impaired blood vessel invasion, cartilage remodeling, and skeletal growth [38–40]. Blood vessels serve as a way of transporting circulating osteoblasts [41] and osteoclast precursors [42] to sites undergoing active remodelling [43]. Cancer metastases to bone cause alterations in normal bone metabolism and the balance between osteoclasts and osteoblasts in favour of one or the other, resulting in destructive lytic or sclerotic lesions, or a combination of both [44]. Osteoclasts are primarily responsible for tumour induced bone destruction, and during the resorption of the bone matrix, embedded growth factors are released that produce a permissive microenvironment and further promote tumour growth [30, 45]. Of note, in human prostate cancer, bone metastases generally favour an osteoblastic phenotype, in contrast to other metastases such as those from renal cell carcinoma, which are often lytic [46, 47].

**Figure 2:**
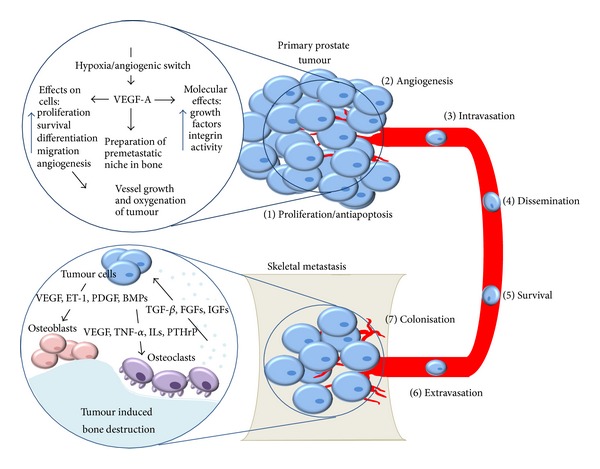
Tumour induced bone destruction. In the prostate, an angiogenic switch promotes the secretion of VEGF, leading to various effects on cells, increasing the release of growth factors and activating integrin activity. (1) Proliferation/antiapoptosis: the cells undergo a transformation that increases the proliferation, survival, differentiation, and migration of the tumour cells. (2) Angiogenesis: the actions of VEGF activate angiogenesis—allowing the tumour cells to access nutrients. (3) Intravasation: cells invade into the local stroma and then enter the local vasculature. (4) Dissemination: cancer cells travel to distant target organs. (5) Survival: Cells can undergo apoptosis or stop proliferation after dissemination and need to evade local immune surveillance. (6) Extravasation: invasion of target organ. (7) Colonisation: after surviving dissemination and extravasation of the target site, tumour cells invade the bone and undergo progressive growth. In metastatic bone disease, tumour cells secrete humoral factors that stimulate osteoclastic and osteoblastic recruitment and differentiation. Once these osteoclasts begin to break down bone, growth factors are released, stimulating growth of the tumour cells. This encourages the tumour cells to release factors that further increase bone resorption by osteoclast and stimulate bone formation through the activation of osteoblasts.

The spread of prostate cancer metastasis to bone is a complex process involving tumour cell migration from the primary tumour site, dissemination through the vascular system, extravasation, and finally establishment, growth, and invasion at the secondary bone site [4]. In a clinical trial of patients with metastatic prostate cancer, bone metastases were noted in 88.9% of patients, compared with soft tissue/lymph node metastases in 22.2% and visceral metastases in 16.7%, demonstrating the preferential homing capabilities of prostate metastasis to bone [48]; however the propensity of prostate cancer to metastasize to bone and the vertebral column remains largely unknown. Prior to the attachment of these cancer cells to bone, it is thought that a premetastatic niche may be created by nonmalignant bone marrow-derived cells that are stimulated by tumour-secreted proteins, which in combination with various bone-enriched growth factors, cytokines, proteases, and components of the extracellular matrix such as a high extracellular calcium concentration support the colonization and growth of prostate cancer cells in bone [49–51]. The actions of VEGF are thought to assist in tumour cell recognition of bone and encourage nesting of the tumour cells in bone [52]. Prior to attachment, VEGF via VEGFR2 modulates the migratory responses of tumour cells encouraging adhesion molecules such as fibronectin and bone sialoprotein within the extracellular matrix [53]. Additionally, VEGF and its cognate receptors may be able to regulate integrin activity, promoting recognition of the bone matrix [32]. Various tumour-expressed growth factors, endothelial markers, and cytokines attract and activate osteoclasts, which in turn disrupt the bone balance through overstimulation and discharge of bone-derived growth factors (Figure 2). Other factors which affect the progression of prostate cancer to bone are Dickkopf-1 (DKK-1), sclerostin, and Wnt signalling. Upregulation of DKK-1 and sclerostin enhances osteoclastic activity by suppressing Wnt signaling and is thought to be able to inhibit the advancement of bone cancer metastases [54, 55]. Whilst DKK-1 levels in patients with bone metastases decrease, Wnt levels rise [56]. This increase of Wnt signalling promotes osteoblast and inhibits osteoclast differentiation, leading to an osteoblastic tumour phenotype [56, 57]. Furthermore, in response to various hormonal, cellular, and cytokine signals, receptor activator of nuclear factor-k B ligand (RANKL), induces osteoclast formation and activation [58, 59]. Osteoprotegerin (OPG) may act as a decoy receptor for RANKL in order to inhibit osteoclastogenesis, which in turn increases osteoblast formation [60]. This RANK/RANKL/OPG axis therefore is important in determining the phenotype of the bone tumour [59]. These factors are deposited into the bone matrix and create a microenvironment that is favourable for cancer cells, leading to further proliferation of tumour cells and bone degradation through the secretion of osteolytic factors [61].

Expression levels of VEGF and VEGFRs have also been shown to be elevated at the site of bone metastases in comparison to primary prostate tumours, indicating that VEGF is an important factor in metastasis development, particularly to bone [53]. Increased VEGF plasma levels have been shown to correlate with skeletal metastasis and poor prognosis in prostate cancer patients and VEGF expression levels in many cancer types have been shown to correlate with poorer prognosis and metastatic potential [62]. However, other studies have shown that there is no correlation between VEGF serum levels and prognosis [63, 64]. The expression of VEGF is upregulated in prostate cancer and is associated with clinical stage, Gleason score, tumour stage, progression, metastasis and survival [65–67]. In prostate cancer VEGF-dependant autocrine stimulation activates the *α*V*β*3 integrin via the VEGFR2 receptor leading to cell proliferation, survival and recognition of extracellular matrix components, which may influence their metastatic capabilities [53]. Many prostate cancer cell lines known to produce osteoblastic metastases highly express VEGF [68].

Interactions of VEGF with markers such as TNF-*α*, IL-6, IL-8, and CCN3 have been linked to the pro-angiogenic activities of tumour cells [69–71]. High expression of VEGF has been observed in human metastatic prostate cancer cell lines PC-3, Du145, and line LNCaP-C4–2, where it has been shown to promote osteoblastic differentiation and activity *in vitro *[53, 71–73].

## 5. Current Treatment of Prostate Cancer and VEGF/VEGF-R Targeted Therapies

Traditionally, androgen ablation has been the main treatment for the prevention of metastases from prostate cancer. As prostate cancer cells are initially dependant on androgens, suppressing the levels of testosterone and dihydrotestosterone decreases the growth rate of prostate cancer cells [74]. However, after this initial response these cells can become castrate-resistant and develop a more aggressive phenotype, with increased VEGF expression and proliferative potential [74, 75]. The commonest conventional treatments for bone metastases secondary to prostate cancer are chemotherapy, radiation, and surgery. Although chemo- and radiotherapy has the potential ability to kill rapidly dividing cancer cells, they each have their own toxic side effects and there is little survival benefit in patients with metastatic cancer [76, 77]. Bisphosphonates such as zoledronic acid or Denosumab, a human monoclonal antibody that targets RANKL signalling, also have a therapeutic role in preventing skeletal-related events in bone metastases via inhibition of osteoclast-mediated bone resorption [78]. Patient morbidity and mortality due to local tumour recurrence, multimetastatic disease, loss of structural function of the bony skeleton destroyed by tumour, and metastatic epidural nerve or spinal cord compression remain important challenges.

Due to the multifaceted effect VEGF has on tumour angiogenesis, tumour cell proliferation, and bone destruction, antiangiogenic therapies targeting the VEGF pathways have shown promising early clinical application and are being investigated in clinical trials. These anti-VEGF therapies consist of VEGF-neutralizing antibodies and tyrosine kinase receptor inhibitors. Bevacizumab is a monoclonal IgG1 antibody that blocks the binding of VEGF-A to its receptors by neutralizing all VEGF isoforms and bioactive proteolytic fragments through the binding of the antibody Fab-ligand epitope to the Gly88 residue of VEGF [79]. Bevacizumab is currently in Phase II clinical trials in relapsed prostate cancer and is approved by the US Food and Drug Administration (FDA) for treatment of metastatic colorectal, renal, and breast cancer and other solid tumours [80, 81]. Similarly, Aflibercept is another antibody which neutralizes VEGF and is currently being used in Phase II clinical trials for patients with recurrent or metastatic urothelial cancer [82]. Tyrosine kinase inhibitors act on VEGF receptors inhibiting activation following ligand binding [83]. Ramucirumab is a human IgG1 monoclonal antibody which binds to the extracellular domain of VEGFR-2 and blocks the VEGF-A to VEGFR-2 interaction and subsequent downstream signaling [84]. Other small molecule tyrosine kinase receptor inhibitors include Semaxanib, which targets a single receptor (VEGFR2), and Sorafenib, which targets multiple tyrosine kinase receptors VEGFR1, -2 and -3, as well as platelet-derived growth factor receptor-*β* [83, 85]. Recently, studies have suggested that using anti-VEGF therapies such as Bevacizumab in concert with radiation therapy or chemotherapy may be able to increase the response to radiation therapy [86]. These synergistic actions have been reported in several preclinical studies and have been shown to improve the survival rates in patients with advanced cancers and decrease levels of radiation necrosis [86–88].

## 6. Conclusion

To date there have been many articles published suggesting the possible molecular mechanisms behind the propensity of prostate cancer to metastasize to bone and the vertebral column. VEGF has been implicated in many of these, including facilitating cancer cell migration to bone, induction of angiogenesis, and stimulating bone forming and resorbing cells of the bone marrow. Anti-angiogenic treatments targeting the VEGF/VEGF receptor pathway have shown promising early clinical application. Further research is required to determine whether this may be translated into better disease control, decreased morbidity, higher survival rates, and improved quality of life in patients with prostate cancer.
